# Impact of prematurity and the CTG repeat length on outcomes in congenital myotonic dystrophy

**DOI:** 10.1186/s13104-020-05186-z

**Published:** 2020-07-23

**Authors:** Yu Saito, Kenta Matsumura, Misao Kageyama, Yuichi Kato, Eiji Ohta, Kiyoaki Sumi, Takeshi Futatani, Taketoshi Yoshida

**Affiliations:** 1 Division of Neonatology, Maternal and Perinatal Center, Toyama University Hospital, Toyama, Japan; 2 Toyama Regional Center for Japan Environment and Children’s Study, University of Toyama, Toyama, Japan; 3 Department of Neonatology, OKAYAMA Medical Center, Okayama, Japan; 4 Department of Neonatology, Anjo Kosei Hospital, Aichi, Japan; 5 Department of Pediatrics, Fukuoka University, Fukuoka, Japan; 6 Department of Pediatrics, Aizenbashi Hospital, Osaka, Japan; 7 Department of Pediatrics, Toyama Prefectural Central Hospital, Toyama, Japan

**Keywords:** Myotonic dystrophy, CTG repeat, Discrimination analysis, Prematurity, Predicting prognosis

## Abstract

**Objective:**

Patients with congenital myotonic dystrophy (CDM) tend to be born preterm. Although the CDM severity generally depends on the CTG repeat length, prematurity may also affect the prognosis in patients with CDM. Given that preterm birth is expected to increase the risk of CDM in newborns, we investigated the outcomes of newborns with CDM according to gestational age to assess prematurity and the CTG repeat length for predicting prognosis.

**Results:**

We assessed the outcomes of 54 infants with CDM using data collected from our hospitals and previously published studies. The patients were divided into mild and severe groups based on clinical outcomes. Logistic regression analysis was performed to estimate odds ratios (ORs) for CDM prognosis according to gestational age and the CTG repeat length and to construct a predictive model. Logistic regression analysis showed both the CTG repeat and gestational age were significantly associated with severe outcomes in patients with CDM (OR: 32.27, 95% CI 3.45–300.7; *p* = 0.002 and OR: 0.73, 95% CI 0.58–0.93; *p* = 0.0094, respectively). This predictive model for CDM prognosis exhibited good sensitivity (63%) and specificity (86%). Both prematurity and the CTG repeat length were significantly associated with the CDM severity.

## Introduction

Myotonic dystrophy type 1 (DM1, OMIM #160900) is an autosomal dominant disorder that affects skeletal and smooth muscles as well as the respiratory, gastrointestinal systems. DM1 is caused by an expansion of cytosine-thymine-guanine (CTG) trinucleotide repeats in the 3′ untranslated region of the dystrophia myotonica protein kinase (*DMPK*) gene [1–4]. DM1 demonstrates anticipation because the CTG repeat expansion in DMPK seems to increase with parental transmission, especially by mother [5]. The current clinical classification of DM1 is based on age at onset and length of the CTG expansion; therefore, DM1 severity depends on the length of the CTG expansion [6–10]. Congenital DM (CDM) is the most severe form of DM1, and neonatal mortality ranges between 16% and 41% [11]. Although the disease severity generally dependent on CTG repeats size, there are wide spectrum of involvements in CDM patients [12, 13]. The cause of death is mainly respiratory distress which may be correlated with the CTG repeat length [6, 8]. The presence of respiratory distress can distinguish between mild and severe CDM [8]; therefore, the respiratory system is a vital organ for predicting prognosis in CDM newborns.

Most CDM newborns inherit DM1 from their mothers. Pregnant women with DM1 have a preterm delivery rate of approximately 30%–50% [8, 14, 15]. Preterm newborns (gestational age less than 37 weeks) and low birth weight infants (less than 2500 g) are generally accompanied by some complications caused by prematurity such as bronchopulmonary dysplasia (BPD) and intestinal movement disorder. Consequently, preterm CDM newborns transmitted especially by mother are assumed to be at a much higher risk for dysfunction of the pulmonary and intestinal systems because they are affected by the double risk factors of CDM and preterm birth. Since we expected preterm birth to increase the burden on CDM newborns, we investigated the outcomes of CDM newborns according to gestational age (prematurity) and the CTG repeat length for predicting prognosis.

## Main text

### Methods

#### Data collection

This retrospective case series collected patients information of newborns diagnosed with CDM in our six NICUs, which are all tertiary hospitals, from January 2002 to July 2019 in Japan. We obtained clinical data including gestational age, birth weight, length of hospital stay, outcome, and the CTG repeat length from the medical charts. In addition, CDM infants from previous literature published between January 2002 and July 2019 were included when at least gestational age, length of hospital stay, outcome, and the CTG repeat length had been reported. This study was reviewed and approved by the Toyama University Review Board (R2019038).

#### Outcome analysis by combining the CTG repeat length and gestational age

Length of hospital stay was used as a marker of CDM severity. CDM patients were classified into the mild group (i.e., a hospital stay of less than 1 year) or the severe group (i.e., death or a long hospital stay of more than 1 year). The choice of long hospital stay as the threshold was completely arbitrary, but Japanese national surveillance showed only 4.7% of newborns of NICU stayed in hospitals more than 1 year [16]. This long-term hospitalization was intuitively meaningful for distinguishing the severity of CDM infants. In case of preterm infants, hospital stay was calculated from expected date of birth. For the analysis of CTG repeat length, we adopted the median value if there were between 1800 and 2200 CTG repeats (2000 repeats in this case) and then applying a logarithmic transformation (log_2_) to normalize the distribution. To investigate the outcomes of CDM infants, a logistic regression analysis was conducted to estimate odds ratio (OR) and its 95% confidence interval (CI) for CDM severity according to log_2_ CTG repeat length and gestational age and to construct a predictive model. A *p* value of less than 0.05 was considered statistically significant, using SAS version 9.4 (SAS Institute Inc., Cary, NC, USA).

## Results

We analyzed data on 22 CDM infants from our hospitals and 31 patients from previous studies [17–33]. We summarized the clinical characteristics of all 53 CDM infants in Table 1. The median gestational age at delivery was 34.6 weeks (range, 23.5 to 42.1 weeks), with a preterm delivery rate of 70.4% (38/54 cases). The median neonatal birth weight was 2085 g (range: 526 to 3600 g), with a low birth weight rate of 68.5% (37/54 cases). The CTG repeat length varied from 600 to 3000 (median 1700). By outcome, the mild group included 37 infants, whereas the severe group included 9 dead infants and 7 infants who required a long hospitalization. The median length of hospital stay in the infants that survived was 80 days (0 to 673).

**Table 1:** Summaries of clinical characteristics of congenital myotonic dystrophy

Case	GA at birth (wk)	Birth weight(g)	No. of CTG repeats	Outcome	Hospital stay (d)
Our cases 1	34	2174	1900	1	134
2	23.5	526	2600	2	65
3	40	3232	1820	1	43
4	36	3096	1700	1	90
5	26	982	2400	2	326
6	36.3	2031	2100	2	3
7	33.6	2085	1200	1	141
8	35.4	2597	2100	2	2
9	34.2	2252	2200	2	409
10	35.5	2370	1000	1	43
11	37.4	2886	1250	1	86
12	37.4	2434	1450	1	100
13	37.6	2226	1800	1	71
14	38.2	2344	1400	1	71
15	30	1190	1000	1	96
16	37	2600	900	1	0
17	37	2676	600	1	2
18	25	758	1100	1	236
19	33.1	2018	2200	2	2
20	33.3	1656	2200	1	129
21	28.3	848	1500	2	285
22	31.3	1640	2400	1	160
Yamaguchi [17]	37.6	2838	1375	1	49
Yee [18]	34.6	2058	2700	2	397
	36.6	2985	1300	1	29
	38	3600	1100	1	20
	38.2	3140	1300	1	21
	29.1	1380	1000	1	260
	38.5	3450	700	1	24
	31.5	1710	1700	1	91
	37	2850	1270	1	5
	31	1640	2000	2	45
Tsuji [19]	30	1322	1600	2	365
	35	2618	2200	2	635
	35	1892	1300	1	180
Yamashita [20]	37.6	2876	2600	1	120
Fuma [21]	31.6	1411	2500	2	730
Banno [22]	37	2786	1100	1	80
	36	2434	1650	1	70
Kanazawa [23]	25.1	678	2100	2	61
Minami [24]	40	2935	1630	1	40
Takagi [25]	35.4	2266	2100	1	166
Sato [26]	42.1	3378	2100	1	123
	26.2	896	1000	1	153
Miyagi [27]	29	1200	1800	2	17
Utunomiya [28]	34.6	1890	2900	1	136
Kondo [29]	23.3	660	1950	2	425
Yanagi [30]	36.2	2410	1500	1	172
	34.4	1724	1000	1	145
	33.3	2014	3000	1	203
Enomoto [31]	33.1	2190	2900	2	545
Saito [32]	28.6	850	1700	1	221
Nakayama [33]	28.5	946	1750	1	240

Outcome 1, discharge within 1 year; 2, death or discharge after 1 year

GA, gestational age

Logistic regression analysis showed that both log_2_ CTG repeat length and gestational age were significantly associated with severe outcome of CDM patients (OR 32.27, 95% CI 3.45–300.7; *p* = 0.002 and OR 0.73, 95% CI 0.58–0.93; *p* = 0.0094, respectively). Discriminant line, on which the probability of an infant will be classified into each outcome is the same, derived using this analysis was following:

1 $$ { \log }_{ 2} {\text{CTG repeat length}}\, = \,0.0 8 90\, \times \,{\text{Gestational age }}\left( {\text{weeks}} \right)\, + \, 8. 1 5 $$

Taking exponential transform of both sides leads to

2 $$ {\text{CTG repeat length}}\, = \, 2^{{(0.0 8 90 \times {\text{ Gestational age }}\left( {\text{weeks}} \right) \, + { 8}. 1 5)}} $$

**Fig. 1:**
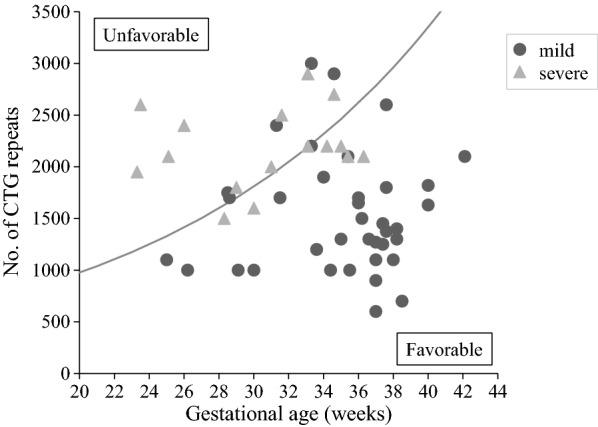
Scatterplot of CDM patients according to the number of CTG repeats and gestational age. Discriminant curve distinguishing between favorable and unfavorable outcomes was derived using a multivariate logistic regression. See text for details

According to this discriminant curve, CDM patients were divided into two groups based on their predictive prognosis: favorable and unfavorable groups (Fig. 1). This equation provided 63% sensitivity, 86% specificity, and a 67% positive predictive value.

## Discussion

The clinical course of CDM in patients assessed in this study varied from one mild case (our case 16) without NICU admission to life-threatening illnesses (Table 1). Throughout this study, we provided a useful predictive prognosis model of CDM patients by combining gestational age and the CTG repeat length (Fig. 1). We revealed that OR decreased by 27% with 1 week increase in gestational age, whereas OR increased by 31.27 with 1 unit increase in log_2_ CTG repeat length, in other words, OR increased by 31.27 whenever the number of CTG repeat length doubled. The current clinical classification of DM1 is based on age at onset and CTG length [7, 12, 13, 34, 35]. On the other hand, several reports have shown no evidence of an effect of CTG repeat length on clinical severity [8, 11, 36]. Since the severity of CDM depends on the status of respiratory complications [6], prematurity is supposed to worsen respiratory status. This discriminant curve increases according to gestational age in Fig. 1; therefore, prematurity is a concern for poor outcomes in CDM newborns. Our results suggest that it is crucial to take into account both prematurity and the CTG repeat length for predicting CDM prognosis.

Interestingly, all term newborns (> 37 weeks) with CDM showed a good prognosis. This may have occurred because these infants had enough time for their respiratory system to mature until term. CDM mortality rates range from 16% to 41% and are generally caused by respiratory insufficiency [11]. Preterm CDM newborns often require mechanical ventilation for a long period of time, which could worsen their lung function. It is important that CDM newborns remain in the uterus for as long as possible. Another reason for this finding is that the term CDM newborns had fewer CTG repeats (median: 1300) than preterm ones (median of 1900). Several studies have indicated the CTG repeat correlated with the disease phenotype [8, 34, 35]. Thus, having few CTG repeats with term CDM newborns induces a favorable outcome. Furthermore, information on GA at birth would be useful to predict the outcomes of preterm CDM newborns.

Approximately 30%–45% of pregnant women with DM1 undergo preterm labor and 17%–25% experience polyhydramnios [14, 15]. In our study population, 68.2% of women had preterm labor (15/22 cases, Table 1) and 68.2% had polyhydramnios (15/22 cases, data not shown). Case No. 3 (CTG repeats: 1820) with polyhydramnios had the amniotic fluid removed twice by amniocentesis at GA 28 weeks (470 ml) and 34 weeks (700 ml). Consequently, this patient was born at 40 weeks and displayed spontaneous breathing without mechanical ventilation. To prolong pregnancy in pregnant women with DM1, amniocentesis may help treat polyhydramnios. These data suggest the possibility of improvement in CDM infants. For example, when evaluating CTG repeat length from 1000 to 2000 repeats in Fig. 1, most patients who were less than GA 32 weeks had unfavorable outcomes. Conversely, patients more than GA 32 weeks all showed favorable outcomes (Fig. 1). These results highlight that the removal of amniotic fluid by amniocentesis may prolong the duration of pregnant mothers with DM1 and may improve outcomes in CDM infants.

## Conclusion

This study showed that both gestational age and the CTG repeat length were associated with outcomes in CDM infants. We can predict the prognosis of CDM fetus or newborns based on gestational age and the CTG repeat length which may be helpful for medical staffs and their parents. Amniocentesis for polyhydramnios in mothers with DM1 may prolong the duration of pregnancy and improve outcomes in CDM infants.

## Limitations

This study has several limitations. First is our data bias. This retrospective case series collected patients information of newborns diagnosed with CDM in our six NICUs, which are all tertiary hospitals in Japan. Our rate of preterm infants was much higher (68.2%) than other reports (30%–45%) [14, 15], which indicates that our hospitals might treat more severe patients. Although we collected many data from literatures as a retrospective case study, there is no significant difference between our patients and literatures’ patients regarding gestational age (mean 33.6wk vs 33.7wk, p = 0.98), CTG repeat length (mean 1673.6 vs 1980.5, p = 0.32), birth weight (mean 2028.2 g vs 2096.8 g, p = 0.76), and hospital length (mean 113.4 days vs 189.6 days, p = 0.09). We believe to have reduced our data bias as little as possible. Second, our definition of severe includes death and a long stay in the hospital of more than 6 months. This length of hospital stay is completely arbitrary. However, this discriminant analysis showed such a high sensitivity and specificity that this time period could be useful for predicting prognosis. Further worldwide studies are necessary to more accurately predict the prognosis of CDM infants.

## Data Availability

All data is confidential regarding patient information.

## Abbreviations

CDM: Congenital Myotonic Dystrophy
CTG: Cytosine-Thymine-Guanine
